# Stearic Acid Coated MgO Nanoplate Arrays as Effective Hydrophobic Films for Improving Corrosion Resistance of Mg-Based Metallic Glasses

**DOI:** 10.3390/nano10050947

**Published:** 2020-05-15

**Authors:** Yonghui Yan, Xiaoli Liu, Hanqing Xiong, Jun Zhou, Hui Yu, Chunling Qin, Zhifeng Wang

**Affiliations:** 1 School of Materials Science and Engineering, Hebei University of Technology, Tianjin 300401, China; 201821803008@stu.hebut.edu.cn (Y.Y.); zhoujun@hebut.edu.cn (J.Z.); yuhuidavid@hebut.edu.cn (H.Y.); clqin@hebut.edu.cn (C.Q.); 2 School of Materials Science and Engineering, Hebei University of Science & Technology, Shijiazhuang 050018, China; iven308@126.com; 3 Department of Mechanical and Electronic Engineering, Changsha University, Changsha 410022, China; xhanqing@ccsu.edu.cn; 4 Key Laboratory for New Type of Functional Materials in Hebei Province, Hebei University of Technology, Tianjin 300401, China

**Keywords:** Mg, metallic glass, hydrophobic film, corrosion resistance

## Abstract

Mg-based metallic glasses (MGs) are widely studied due to their high elasticity and high strength originating from their amorphous nature. However, their further application in many potential fields is limited by poor corrosion resistance. In order to improve this property, an MgO nanoplate array layer is first constructed on the surface of Mg-based MGs by cyclic voltammetry (CV) treatments. In this situation, the corrosion resistance and hydrophilicity of the material are enhanced. Then, stearic acid (SA) can effectively adhere onto the surface of the MgO layer to form a superficial hydrophobic film with a water contact angle (WCA) of 131°. As a result, the SA coated MgO hydrophobic film improves the corrosion resistance of Mg-based MGs in 3.5 wt.% NaCl solution obviously. In addition, the effects of four technological parameters (solution concentration, sweep rate, cycle number, and reaction temperature) in the CV process on the morphology and size of nano-products are investigated in detail. The work proposes a new method for the creation of nanostructures on the surface of materials and provides a new idea to increase the corrosion resistance of MGs. The related method is expected to be applied in wider fields in future.

## 1. Introduction

Magnesium alloys are the lightest metal structural materials, which possess the characteristics of high specific stiffness, high specific strength, good shock absorption, good electromagnetic shielding, as well as easy processing and recovery. Magnesium alloys receive broad research interest across many fields including the automotive industry, aerospace, electronic communications, and biomedicine [1,2,3,4,5]. Unfortunately, the poor corrosion resistance of magnesium alloys limits their further applications, especially in coastal areas. That is because elemental Mg presents high chemical activity and low electrochemical potential in liquid environments containing chloride ions [6,7], leading to an etch towards Mg from the alloy. Mg-TM-RE (TM: transition metal such as Zn, Cu, Ni, and RE: rare-earth metals such as Nd, Yb, or Y) metallic glasses (MGs) exhibit better compressive strength and corrosion resistance than crystalline state alloys with the same composition due to their uniform and long range disordered structure [8,9,10,11,12]. In recent years, many studies related to Mg-TM-RE MGs have been carried out, but the corrosion resistance of Mg-based MGs still represents a key issue to be addressed and improved in order to meet the demand of the market [13].

The existing works show some effective strategies to improve the corrosion resistance of Mg-based MGs. Adjusting the element composition and proportion of the MGs [14,15,16] is one of the simple strategies. By introducing iron particles into Mg-based MGs [17] and by forming a micro-arc oxidation (MAO) layer containing Si on the surface of Mg-based MGs [18], the corrosion resistance of the MGs can also be improved. Moreover, the preparation of hydrophobic film (a water contact angle of more than 90°) on the surface of materials is also an effective method to enhance the corrosion resistance [19,20,21]. The hydrophobic surface is constructed by introducing low surface energy materials onto the sample surface to prevent the corrosive ions from etching the alloy. In this way, the anti-corrosion ability of the sample can be improved. The reported methods for synthesizing hydrophobic films include the hydrothermal method [22], ball milling [23], flashlight irradiation [24], multi-arc ion plating [25], hydrolysis co-precipitation method [26], etc. Many existing methods are more costly and complicated [27,28], so it is necessary to develop a new method with lower cost and more convenient operation. In addition, both MgO protection layers and stearic acid layers are reported to effective in improving corrosion resistance of Mg alloys [29,30,31]. The research on the combination of the two protective layers is still lacking.

In this study, the MgO nanoplate array is fabricated on the surface of Mg_66_Zn_30_Yb_4_ MGs by cyclic voltammetry (CV) and dehydration treatment for the first time. The stearic acid (SA) coated MgO composite hydrophobic film is then synthesized by immersing the sample into SA to enhance the corrosion resistance of the Mg_66_Zn_30_Yb_4_ MG significantly. The treatment technology developed in this work provides a new idea to synthesize various nanostructures on the surface of materials, and also provides a simple method for improving the corrosion resistance of MGs, establishing a research foundation for the further application of Mg-based MGs in corrosive environments.

## 2. Materials and Methods

### 2.1. Preparation of Glassy Ribbons

Mg, Zn, and Yb metallic ingots (>99.9 wt.%) were melted into a button-type master alloy ingot with nominal composition of Mg_66_Zn_30_Yb_4_ (at %) by arc-melting method [32]. The ingot was melted three times to ensure a uniform composition. Melt-spinning method [33] was adopted to spray the remelted master alloy melts from the small hole at the tip of a quartz tube onto the high-speed rotated copper roller (1500 r/min). Finally, the Mg_66_Zn_30_Yb_4_ glassy ribbon (GR) [34] 2 mm wide, 30 μm thick, and dozens of centimeters long was produced.

### 2.2. Preparation of Hydrophobic Surface

**Figure 1:**
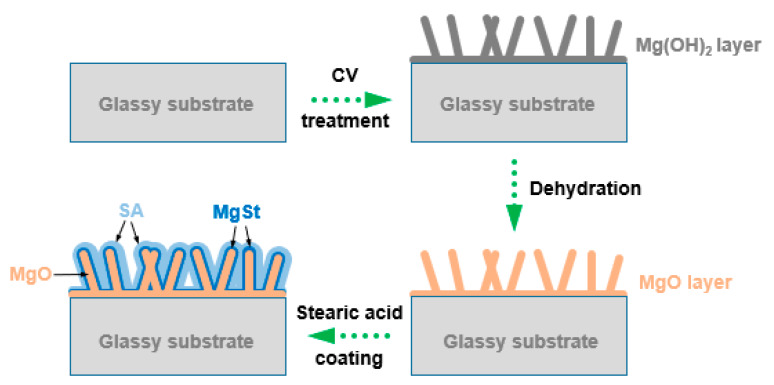
Schematic illustration showing the synthesis procedure of the stearic acid coated MgO hydrophobic film on the Mg_66_Zn_30_Yb_4_ glassy ribbon (GR).

Firstly, CV treatment was performed through the electrochemical workstation to construct the Mg(OH)_2_ micro/nanostructure on the surface of the Mg_66_Zn_30_Yb_4_ GR. The GR, platinum plate, and Ag/AgCl electrode were used as working electrode, counter electrode, and reference electrode, respectively. The CV cycling was carried out in the voltage range of 1~3 V with a scan rate of 0.01 Vs^−1^ in 1 M KOH electrolytes at 25 °C. Several experiments were carried out in order to investigate the influence of electrolyte concentration, scanning rate, cycle number and experimental temperature on the morphology and size of the Mg(OH)_2_ products. These experiments were made to individuate the more appropriate process parameters before the synthesis of the SA layer. After the construction of the Mg(OH)_2_ layer, the sample was cleaned with deionized water and dried at 60 °C in an oven for 1 h. In this situation, MgO can be formed by dehydration of Mg(OH)_2_. Then MgO coated glassy ribbon was immersed into a 0.01 mol L^−1^ stearate ethanol solution at 50 °C for 5 min. Finally, the sample was removed and dried in an oven at 60 °C for 1 h to obtain a SA coated MgO composite hydrophobic film on the surface of the Mg_66_Zn_30_Yb_4_ GR. The preparation process of the film is shown in Figure 1.

### 2.3. Characterization

Morphological characterization of samples produced with different process parameters were observed by scanning electron microscope (SEM, Nova NanoSEM 450, Nebraska Center for Materials and Nanoscience, Lincoln, NE, USA) with back-scattered electron (BSE) mode (1.00 kV). The sizes of a large number of nanoplates obtained under different process parameters were measured by Nano Measurer V1.2, software developed by Fudan University, Shanghai, China.

The phase composition, the variation in elemental contents with the sample depth, the magnified microstructure, the element composition and valence state of surface MgO layers were characterized by X-ray diffraction (XRD, Bruker, Billerica, MA, USA, D8-advance), SEM-EDS (energy disperse spectroscopy) with line scanning mode, transmission electron microscope (TEM, JEOL JEM-2010FEF, JEOL, Boston, MA, USA) and X-ray photoelectron spectroscopy (XPS, Microlab 350, MMT, LLC., Denver, CO, USA).

Characterizations of the SA coated specimen were revealed by SEM (BSE mode), TEM and the water contact angles (WCA) tests. The WCA were measured at room temperature through a contact angle measuring instrument (Dataphysics OCA20, Filderstadt, Germany). The volume of one drop of deionized water used in the measurement was 4 μL, and the measurement started after the water drop staying on the surface of the film for 1 min.

The superficial morphologies of the experimental samples after immersion were observed by SEM (BSE mode for SA treated specimen, SE mode for original and crystalline state Mg_66_Zn_30_Yb_4_ sample) and EDS (line scanning mode).

### 2.4. Corrosion Resistance and Immersing Test

Polarization curve and open circuit potential tests were carried out to compare the corrosion resistance of different materials. A three-electrode system was adopted in the tests. The CV treated samples, platinum plate, and Ag/AgCl electrode were used as working electrode, counter electrode, and reference electrode, respectively. The potentiodynamic polarization curves were measured in the 3.5 wt.% NaCl solution at a scanning rate of 0.01 V/s. In order to ensure the accuracy of the experiment, the test area of the working electrode was limited in the defined range of 0.4 cm^2^ (0.2 cm × 2 cm). The samples were put into the solution to stabilize for 5 min before testing. For immersing tests, the original GR, crystalline state Mg_66_Zn_30_Yb_4_ alloy and SA treated GR were immersed into 3.5 wt.% NaCl solution at 25 °C. After immersing for 10 min, the surface morphology, cross-sectional morphology, and element distribution of the film were detected.

## 3. Results and Discussion

**Figure 2:**
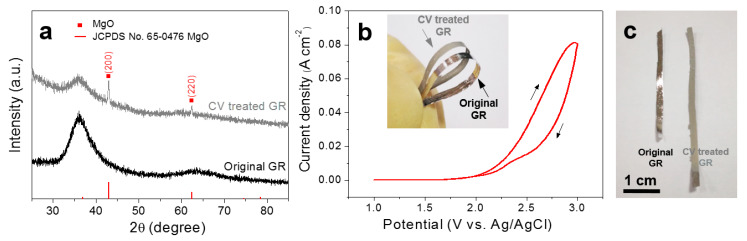
(**a**) X-ray diffraction (XRD) pattern; (**b**) cyclic voltammogram (CV) treatment towards a GR; the inserts: digital photo of GRs with good bendability; (**c**) digital photo of GRs before and after CV treatment.

Figure 2a shows the XRD patterns of the original Mg_66_Zn_30_Yb_4_ GR and the GR after CV and dehydration treatment. The original GR shows typical diffuse-scattering peaks (broad peaks centered at about 36°) [35], indicating an amorphous nature [36]. The amorphous structure of the ribbon after CV treatment is partially preserved, accompanied by additional clear crystalline peaks which corresponds to the (200) and (220) diffraction peaks of MgO (JCPDS No. 65-0476). Figure 2b shows the CV process of the GR with a scan rate of 0.01 Vs^−1^ in 1 M KOH solution at 25 °C (5th lap). During the CV process, the potential changes in the range of 1~3 V, playing a similar role of anodic oxidation. For macro-morphology and bendability testing, GRs in 3~5 cm long were used. The surface color of the ribbon changes from bright silver-white to light gray after CV treatment (Figure 2c), indicating the GR is covered with a layer of products. Moreover, the insert in Figure 2b shows that the Mg_66_Zn_30_Yb_4_ GR still maintains good flexibility after CV treatment, and can be bent continuously for 180°. This provides more possibilities for the application of the material in broader fields.

**Figure 3:**
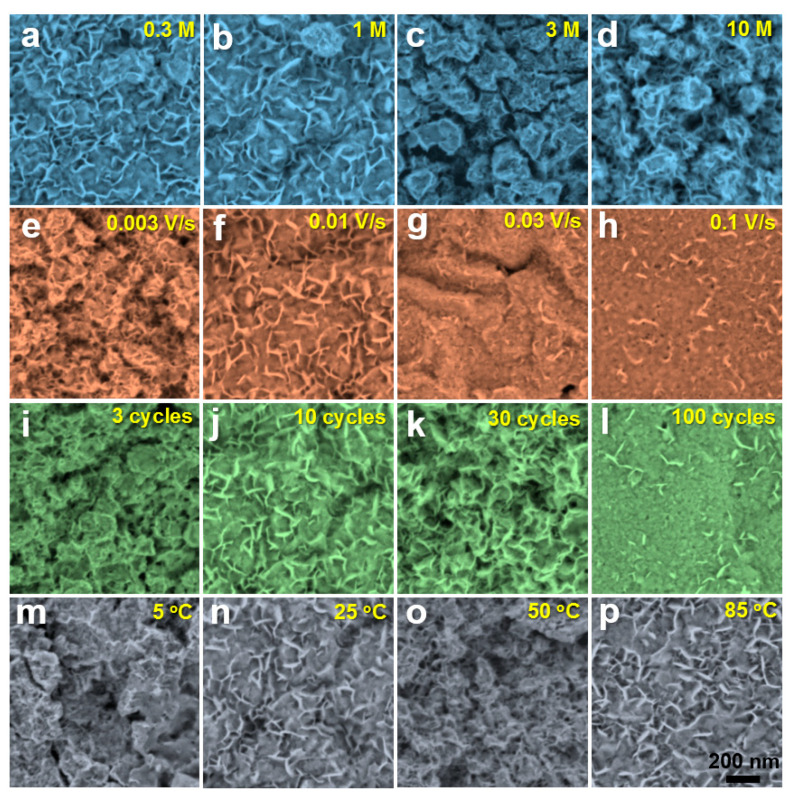
Back-scattered electron (BSE) images of GRs treated by CV under different technological conditions: (**a**–**d**) different KOH solution concentrations, with a scan rate of 0.01 Vs^−1^ at 25 °C for 10 cycles; (**e**–**h**) different sweep rates, in 1 M KOH solution at 25 °C for 10 cycles; (**i**–**l**) different cycle numbers, with a scan rate of 0.01 Vs^−1^ in 1 M KOH solution at 25 °C; (**m**–**p**) different reaction temperatures, with a scan rate of 0.01 Vs^−1^ in 1 M KOH solution for 10 cycles.

Figure 3 shows BSE images towards GR surface after CV and dehydration treatment under different process parameters, including solution concentration, scanning rate, cycle number and reaction temperature. It can be seen that the sample surface presents some typical morphologies such as nanoplate and nanosphere. When the concentration of KOH solutions is low, the MgO nanoplates distribute evenly. With the increase in solution concentration, the nanoplates gradually aggregate into nanospheres (Figure 3a–d). When the scanning rate increases from 0.003 V/s to 0.01 V/s, the size of nanoplates enhances. At the same time, the distribution of nanoplates changes from aggregation to dispersion. When the scanning rate is more than 0.03 V/s, the number of nanoplates is greatly reduced and they become sparser with each other (Figure 3e–h). As the cycle number increases from 3 to 30, the surface nanoplates become more and more dense. However, when the cycle number reaches to 100, the surface nanoplates fall off from the matrix and only a small amount remains on the surface of the material (Figure 3i–l). When the temperature is relatively low (5 °C), a very small number of nanoplates are formed on the sample surface. When the temperature rises to 25 °C or above, the dynamic process of the nanoplate formation is accelerated, and the substrate surface is filled with nanoplates (Figure 3m–p). The possible reasons for the above phenomenon are explained as follows. The higher solution concentration increases the yield of the product, causing aggregating of the nanoplates into nanospheres. The faster scanning speed reduces the reaction time, resulting in a sharp decrease in the yield of nanoplates. Too many reaction cycles increase the yield of the product, but may result in the separation of nanoplates from the matrix. Too low a reaction temperature reduces the velocity of ions and impedes the kinetic process of the reaction, which is not conducive to the synthesis of the products. Based on the above results, it can be seen that the process parameters of the CV treatment demonstrate a great influence on the surface morphology and yield of nanoplates.

**Figure 4:**
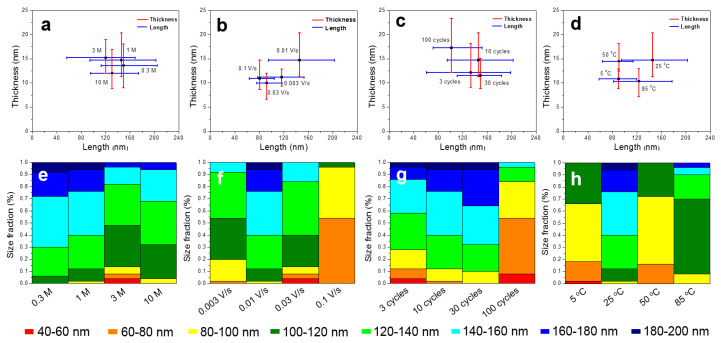
(**a**–**d**) The size statistics of MgO nanoplates obtained under different experimental parameters; (**e**–**h**) fractions of MgO nanoplates with different sizes obtained under different experimental parameters; (**a**,**e**): solution concentrations; (**b**,**f**): sweep rates; (**c**,**g**): cycle numbers; (**d**,**h**): reaction temperatures.

Figure 4 shows the size and proportion statistics of the nanoplate generated under different experimental parameters. It is found that the scanning rate and reaction temperature has a greater influence on the size of nanoplates. For different solution concentrations and cycle numbers, the size differentiation towards the nanoplates is low. In addition to individual process conditions, the nanoplates with a length of 120–180 nm occupy a major proportion in the size statistics. After a number of experiments and data analysis, the stable nanoplate array structure, obtained under the process parameter of 25 °C, 1 M KOH, 0.01 V/s sweep speed for 10 cycles, is selected for subsequent experiments. That is because under these experimental conditions, the uniform nanoplate array structure can be obtained, which is in favor of improving hydrophilicity to further reaction with SA.

**Figure 5:**
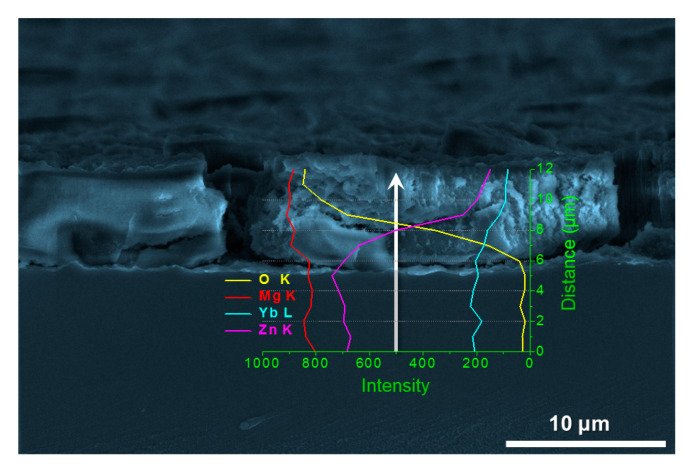
SEM image of cross section of the sample and the corresponding EDS line scanning result.

The SEM image of cross section of the sample prepared under selected parameters is shown in Figure 5. The nanoplate layer is about 5 μm in thickness with a porous and loose structure, which is conducive to the infiltration and attachment of SA. The line scanning result from the substrate to the nanoplate layer shows that the content of O element in the substrate is almost zero, and it increases dramatically after the line scanning reaches to the nanoplate layer. However, the contents of Zn and Yb elements decrease rapidly. The closer to the sample surface, the higher the O content and the lower the Zn and Yb content. Compared with the substrate, the content of Mg element in the film increases slightly, while the contents of Zn and Yb element decrease greatly. The above experimental results further confirm that the material surface is mainly composed of magnesium oxides.

**Figure 6:**
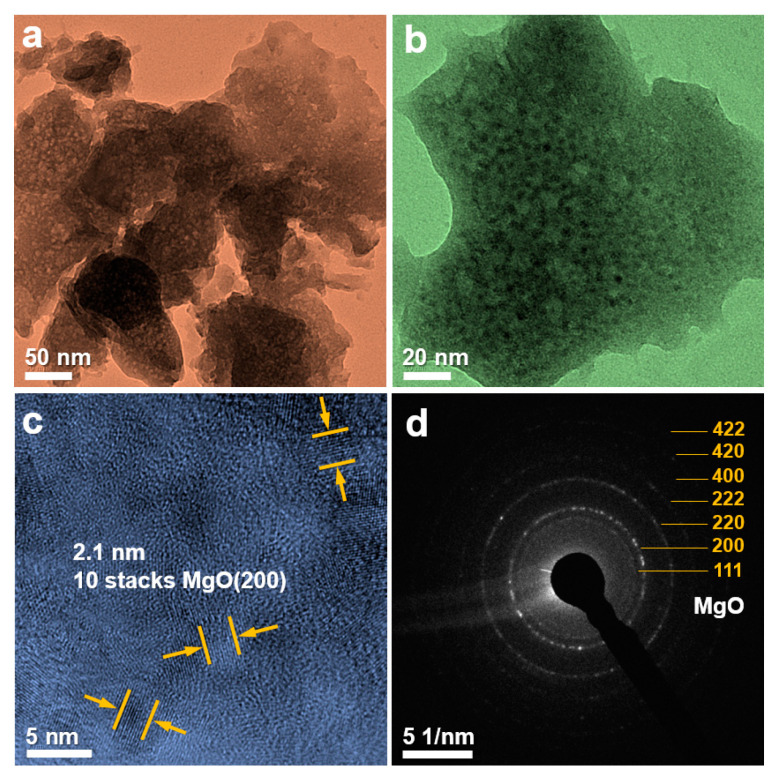
(**a**–**c**) Transmission electron microscope (TEM) images of MgO nanoplates; (**d**) selected area electron diffraction (SAED) pattern of a nanoplate.

In order to better understand the morphology and structure of the nanoplate, the sample is characterized by TEM as shown in Figure 6. Figure 6a,b show the morphology of the nanoplate, which is consistent with the SEM result. Figure 6c is a high-resolution transmission electron microscopy (HRTEM) image showing the measured lattice fringe spacing of 0.210 nm, corresponding to the (200) plane of MgO. The selected area electron diffraction (SAED) diagram of the nanoplate (Figure 6d) shows a polycrystalline concentric ring feature, corresponding to the (111), (200), (220), (222), (400), (420), and (422) crystal planes of MgO. TEM detection further confirms the successful synthesis of MgO.

**Figure 7:**
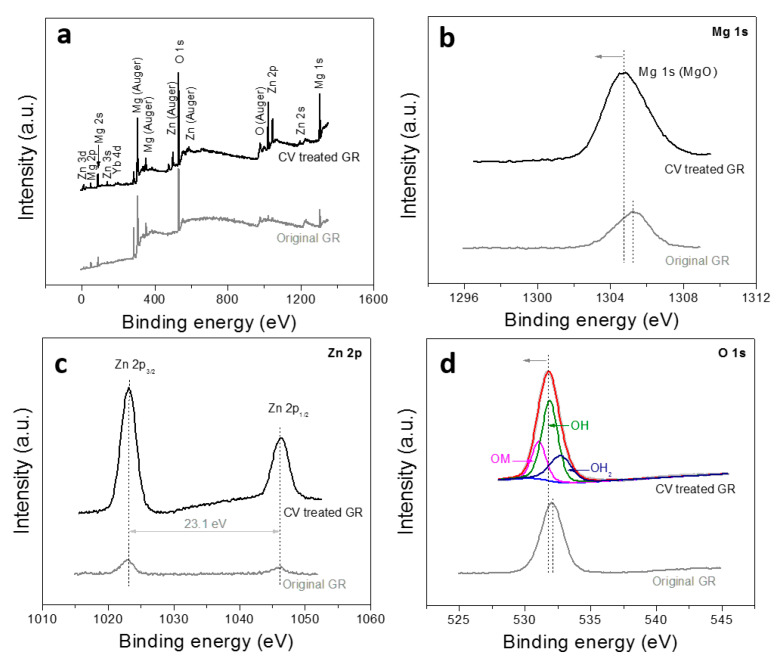
XPS spectra of the nanoplate film: (**a**) fully scanned spectra; (**b**) Mg 1s spectra; (**c**) Zn 2p spectra; (**d**) O 1s spectra.

XPS is used to detect the elemental composition and valence state on the surface of nanoplate films. The fully scanned spectra (Figure 7a) shows that Mg, Zn, Yb and O peaks can be detected on the sample surfaces without other impurities. The high resolution XPS spectrum of Mg 1s is analyzed in Figure 7b. The Mg 1s peak position shifts from 1305.2 eV to about 1304.5 eV after the CV treatment, which is consistent with the previously reported peak position of MgO [37]. In Figure 7c, there are two peaks in the Zn 2p spectra. The difference in binding energy between Zn 2p^3/2^ (1021.8 eV) and Zn 2p^1/2^ (1044.9 eV) is about 23.1 eV, which is in accord with the reported value of ZnO [38], indicating that the nanoplate layer also contains a small amount of ZnO. In addition, the major peak of the O 1s (Figure 7d) can be decomposed into three peaks at 529.8 eV, 531.3 eV, and 532.1 eV, corresponding to OM, OH, and OH_2_ peaks respectively [39,40], which are derived from metal oxides (MgO and ZnO), hydroxides and surface water absorbed in the environment, respectively. The XPS detection demonstrates that the material surface is mainly composed of MgO and a small amount of ZnO as well as metal hydroxides (un-dehydrated hydroxides or formed by the reaction between oxides and water).

**Figure 8:**
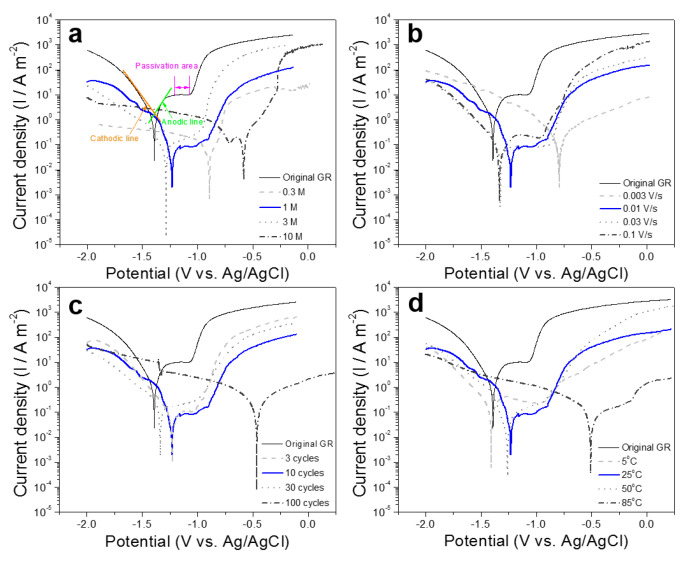
Potentiodynamic polarization curves of samples tested in 3.5 wt.% NaCl solution under different experimental parameters: (**a**) solution concentrations; (**b**) sweep rates; (**c**) cycle numbers; (**d**) reaction temperatures.

The effect of MgO nanoplate layers on the corrosion resistance of Mg-based MGs is studied by potentiodynamic polarization curve in 3.5 wt.% NaCl solutions as shown in Figure 8. The original GR shows a certain range of passivation region. When the MgO nanoplate array layer is coated on the surface of GRs, the corrosion potential and the self-corrosion current of the materials are reduced under most of testing conditions in this paper. In particular, the self-corrosion current can decrease by about two orders of magnitude, indicating that the construction of MgO nanoplate layers can effectively improve the corrosion resistance of Mg-based MGs in NaCl solutions. By comparing the polarization curve positions under different conditions, the technological parameter performed by the blue curve (25 °C, 1 M KOH, 0.01 V/s sweep speed for 10 cycles) is selected in this paper for follow-up experiments. Compared with samples with a greater improvement in corrosion resistance, the material treated by selected technological parameter exhibits a wider passivation area and makes the experimental process more time-saving and easier to implement (no need for high temperature, high solution concentration and CV treatment for multiple cycles).

**Figure 9:**
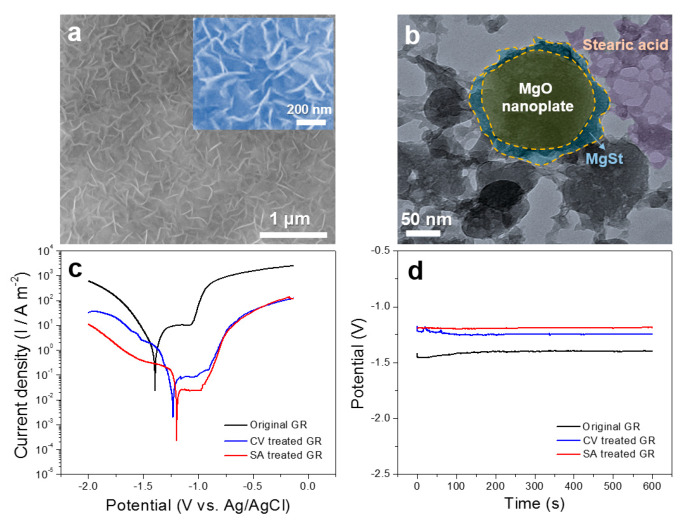
(**a**) BSE image and (**b**) TEM image of the hydrophobic film; (**c**) polarization curves and (**d**) open circuit potential of samples treated in different ways.

Figure 9a shows the surface SEM morphology of the Mg_66_Zn_30_Yb_4_ GR after immersing in SA. The nanoplate array maintains the original morphology after immersion, covered by some flocculent SA. The TEM image (Figure 9b) shows that the surface of the nanoplate is covered with a layer of magnesium stearate (MgSt) and the outermost layer is covered with porous SA. The related reaction is shown in Equations (1) and (2) [41,42], revealing that the reaction between SA and MgO as well as the reaction between SA and Mg(OH)_2_ will form a [CH_3_(CH_2_)_16_COO]_2_Mg (MgSt) interlayer between the two reactants. The nanoplates with good hydrophilcity, distributed in a form of porous networks, are very conducive to the diffusion of SA onto their surfaces, resulting in the formation of a composite hydrophobic layer.

MgO + 2CH_3_(CH_2_)_16_COOH → [CH_3_(CH_2_)_16_COO]_2_Mg + H_2_O (1)

Mg(OH)_2_ + 2CH_3_(CH_2_)_16_COOH → [CH_3_(CH_2_)_16_COO]_2_Mg + 2H_2_O (2)

Figure 9c shows the potentiodynamic polarization curves of the original GR, CV treated GR, and SA treated GR in 3.5 wt.% NaCl solutions. The SA treated GR shows the lowest self-corrosion current and the highest corrosion potential among the three samples, indicating that the corrosion resistance of the CV treated GR is further improved by the coverage of SA. It can be seen from Figure 8d that the open circuit potential (OCP) of the GR increases from −1396 mV to −1246 mV after the CV treatment, while it further increases to −1197 mV after the SA treatment. The increase in OCP (Figure 8d) and the decrease in self-corrosion current (Figure 8c) mutually support that the corrosion resistance of the SA treated GR improves.

**Figure 10:**
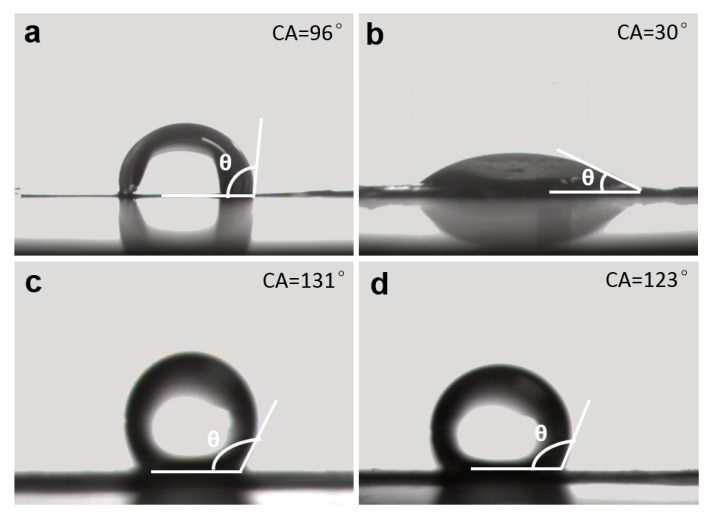
Images of water contact drops for different samples: (**a**) original GR; (**b**) CV treated GR; (**c**) SA treated GR (**d**) SA treated GR after immersion in 3.5 wt.% NaCl for 24 h.

The wettability of different materials can be compared by measuring the water contact angle (WCA). If the contact angle is more than 90°, the surface is regarded as hydrophobic, while if the contact angle is less than 90°, the surface is regarded as hydrophilic [42]. As shown in Figure 10a, the WCA of the original GR surface is 96°, indicating that the original material possesses certain hydrophobicity. After CV treatment, the WCA of GR changes to 30° (Figure 10b), indicating that the material presents good hydrophilicity in this situation, which is ready for the good adhesion of SA on the MgO surface. After SA immersion, the WCA of GR increase to 131° (Figure 10c), showing significantly improved hydrophobicity. Moreover, after immersing in 3.5 wt.% NaCl solution for 24 h, the WCA can still be kept at about 123°, showing good hydrophobic characteristic and good maintenance feature. The above phenomena confirm that the SA treated MgO film has good hydrophobicity and can protect the internal sample effectively for a long time.

**Figure 11:**
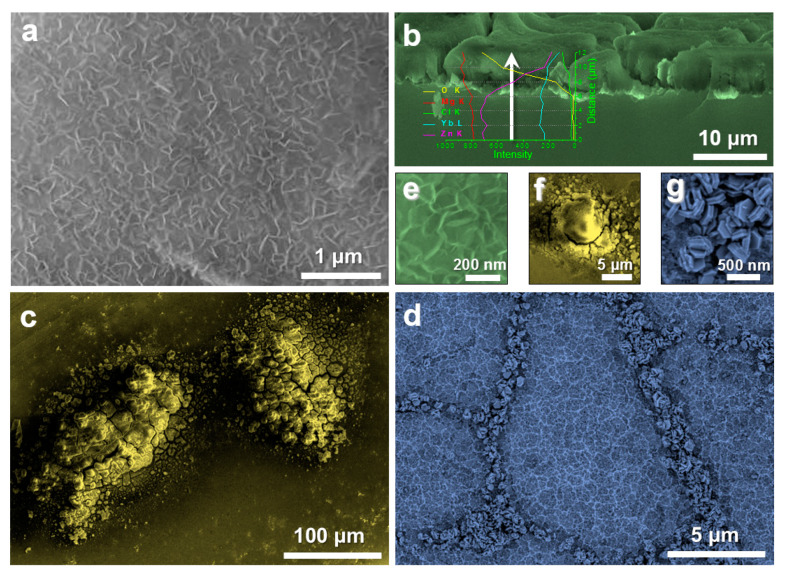
SEM images showing superficial morphologies of the experimental samples after immersion in 3.5 wt.% NaCl solution for 10 min: (**a**) SA treated Mg_66_Zn_30_Yb_4_ GR; (**b**) cross section of the hydrophobic film and the EDS line scanning; (**c**) original Mg_66_Zn_30_Yb_4_ GR; (**d**) crystalline state Mg_66_Zn_30_Yb_4_ sample; (**e**–**g**) enlarged images of (**a**,**c**,**d**) respectively.

Figure 11 presents superficial SEM morphologies of the experimental samples after immersion in 3.5 wt.% NaCl solution at 25 °C for 10 min. Figure 11a shows that the nanoplate array structure on the SA treated GR remains good after corrosion, with no obvious change in morphology (Figure 11e), showing the good effect of the film in improving corrosion resistance. Cross-sectional SEM image (Figure 11b) shows that the thickness of the hydrophobic film after corrosion does not change much and remains in about 5~6 μm. The linear scanning spectrum shows that the content of Cl element in the film is very low, indicating that the film plays an effect role to protect the substrate from corrosion. Figure 11c,f reveals that the obvious surface pitting of the original GR takes place during corrosion, indicating that, although the original GR presents relatively good corrosion resistance, local corrosion still exists. When the crystalline Mg_66_Zn_30_Yb_4_ material is corroded, a certain degree of corrosion occurs inside the grain, forming a porous network. At the same time, a large number of hexagonal nanoplates generate at the grain boundary (Figure 11g), indicating that the crystalline state Mg_66_Zn_30_Yb_4_ material possesses a very general corrosion resistance and is very easy to be corroded. Supplementary Video S1 shows a video comparing the macroscopic corrosion phenomenon of the original GR and the SA treated GR during the corrosion process in a more extreme corrosion environment (a mixture of 20 mL 3.5 wt.% NaCl solution and 0.5 mL 1 M HCl solution). It is found that in such a corrosive environment, a large number of bubbles appear immediately on the surface of amorphous GR when it is put into the corrosive solution, indicating that a drastic reaction occurs on the surface of the sample. However, after the SA treated GR is added to the corrosive solution, there is no obvious corrosion visible to the naked eye on the material surface. Based on the above analysis, the hydrophobic film synthesized in this paper presents a good protective effect to the glassy substrate and can effectively improve the corrosion resistance of Mg-based MGs in Cl ion containing solutions.

## 4. Conclusions

By utilizing the CV treatment method, the MgO nanoplate array films are synthesized on the surface of Mg_66_Zn_30_Yb_4_ GRs successfully. Four CV treated parameters, including solution concentration, scanning rate, cycle number, and reaction temperature, are examined to analyse their effects on the sizes and shapes of the nano-products. It is found that the scanning rate and reaction temperature have a greater influence on the size of nanoplates. In addition, the nanoplates present a main length of 120~180 nm under the experimental conditions. The stable nanoplate array structure, obtained under the process parameter of 25 °C, 1 M KOH, 0.01 V/s sweep speed for 10 cycles, is selected for surface SA modification. As a result, the SA coated MgO composite hydrophobic film with a WCA of 131° is prepared on the surface of the Mg_66_Zn_30_Yb_4_ GR. The composite shows improved corrosion resistance compared with the original amorphous alloy and the crystal material with the same composition. The work provides us a simple, low-cost, energy-saving, and environmentally friendly CV treatment method to create surface nanostructures and improve the corrosion resistance of the Mg_66_Zn_30_Yb_4_ GR, which may promote the development and application of the CV treatment method and is expected to lead to the synthesis of different films with various nanostructures.

## Supplementary Materials

The following are available online at https://www.mdpi.com/2079-4991/10/5/947/s1, Video S1: Macroscopic corrosion phenomenon of the GRs.
